# Mental illness and pulmonary tuberculosis: a bidirectional two-sample Mendelian randomization study

**DOI:** 10.3389/fpsyt.2024.1345863

**Published:** 2024-04-29

**Authors:** Xing Chen, Fengbo Yang, Ronghui He

**Affiliations:** ^1^ Department of Infection, Nanchong Central Hospital, The Second Clinical Medical College, North Sichuan Medical College, Nanchong, Sichuan, China; ^2^ Department of Otolaryngology, Affiliated Hospital of North Sichuan Medical College, Nanchong, Sichuan, China

**Keywords:** mental illness, major depressive, anxiety disorder, bipolar disorder, schizophrenia, pulmonary tuberculosis, Mendelian randomization

## Abstract

**Background:**

Observational studies have confirmed that mental illness and pulmonary tuberculosis are closely related and increase each other’s incidence; however, whether there is a causal genetic association between the two diseases remains unknown. We attempted to answer this question using bidirectional two-sample Mendelian randomization (MR) in a large cohort study.

**Method:**

We performed a bidirectional MR analysis between mental illness (major depressive, anxiety disorder, bipolar disorder, and schizophrenia) and pulmonary tuberculosis using summary statistics from genome-wide association studies in European individuals. The inverse-variance weighted method was used as the primary analytical method to assess causality. In addition, other additional MR methods (weighted median, MR–Egger, and weighted mode) were used to supplement the inverse-variance weighted results. Furthermore, several sensitivity analyses were performed to assess heterogeneity, horizontal pleiotropy, and stability.

**Result:**

We identified no causal genetic association between mental illness and pulmonary tuberculosis after applying the inverse variance weighted method (major depressive: odds ratio (OR) = 1.00, 95% confidence interval (CI) = 0.59–1.71, *P* = 0.98; anxiety disorder: OR = 1.72, 95% CI = 0.05–67.67, *P* = 0.76; bipolar disorder OR = 0.89, 95% CI = 0.66–1.22, *P* = 0.48; and schizophrenia: OR = 1.05, 95% CI = 0.91–1.20, *P* = 0.51). Similarly, pulmonary tuberculosis was not caustically associated with mental illness (major depressive: OR = 1.01, 95% CI = 1.00–1.02, *P* = 0.17; anxiety disorder: OR = 1.00, 95% CI = 0.99–1.01, *P* = 0.06; bipolar disorder: OR = 1.02, 95% CI = 0.98–1.07, *P* = 0.38; and schizophrenia: OR = 1.01, 95% CI = 0.97–1.05, *P* = 0.66).

**Conclusion:**

Our research does not support a bidirectional causal association between the aforementioned mental illnesses and pulmonary tuberculosis.

## 1 Introduction

Tuberculosis (TB), a chronic inflammatory disease resulting from *Mycobacterium tuberculosis* (MTB) infection, can be transmitted via the respiratory tract. The World Health Organization’s data for 2022 reveal that there were approximately 10.6 million individuals with TB in this year, with approximately 1.3 million TB-related deaths recorded. Globally, TB was the second leading cause of infection-related deaths after COVID-19 in 2022 (1). It poses a significant threat to human life and well-being. Similarly, mental illness is also a significant public health concern. Statistics reveal an alarming observation that over 970 million individuals were affected by mental illnesses worldwide in 2019, with anxiety and depression emerging as the most prevalent conditions (2). Unfortunately, a substantial majority of these individuals lack access to adequate treatment and care, thus exacerbating their condition. Moreover, individuals with a mental illness often encounter stigmatization, discrimination, and infringement of their fundamental human rights. TB and mental illnesses are both serious public health concerns, and numerous studies have reported their concurrent incidence in individuals. In particular, psychiatric morbidities are reportedly common in patients with TB (3–6). According to Dasa et al., the incidence rate of depression among people with pulmonary TB in Eastern Ethiopia was notable at 51.9% (7). Similarly, Koyanagi et al., who collected such statistics from 48 countries with low and middle incomes, reported this incidence rate to be 23.3% (8). Furthermore, Doherty et al. also found the incidence rate of mental illnesses in TB patients to be alarmingly high at 70% (9). TB patients with a mental illness are at higher risk for poor healthcare-seeking behavior and medication adherence, which ultimately leads to higher morbidity, mortality, drug resistance, and persistent disease transmission (10). This poses a huge challenge for TB prevention and intervention, particularly in countries with a high burden of TB or mental illness.

The relationships between TB and mental health are highly complex, with the TB–depression comorbidity being widely considered a “syndemic” due to the bidirectional synergy involved (11). This has also aroused great interest among scholars in the potential causes of coexistence of TB and mental illness. Some scholars have found the high incidence of depression and anxiety in TB patients to be closely related to physical disorders, social stigma, and inadequate social support (12). Another study has shown that some anti-TB drugs (e.g., isoniazid and cycloserine) can induce depression, anxiety, or mental illness (9). Moreover, TB also can invade the central nervous system and trigger neurologic symptoms (13). Most importantly, in terms of pathological mechanisms, the reason underlying the association between mental illness and TB may be related to immunity. When the body is subjected to various physiological shocks or psychological stresses, numerous inflammatory factors reportedly reach the damaged area, activate various immune responses, and induce mental illness (14). Similarly, the release of a large number of inflammatory factors and immune imbalances can exacerbate TB (15). In addition, mental illness, including depression, is associated with immune decline, thus potentially increasing susceptibility to TB (11, 16). These studies have found psychosocial stressors to be related to TB-related immune biomarkers, and the existence of these reasonable immune mechanisms seems to support a causal association between mental illnesses and pulmonary tuberculosis (17).

However, most of the current research has been based on observational epidemiological designs, which are likely to have confounding factors that cannot be corrected for. As only evidence can confirm or rule out a possible link between mental illness and TB, the causal link between them remains unclear. Typically, the gold standard for determining causality is randomized controlled trials (RCTs), wherein subjects are randomly divided into a control group and an experimental group to study the effects of a factor. However, in practice, RCTs are very difficult to complete, require a lot of manpower and resources, and sometimes, owing to ethical issues, research on a certain factor can be almost impossible. Mendelian randomization (MR) studies, similar to RCTs, can be conducted using single nucleotide polymorphisms (SNPs) and genome-wide association studies (GWAS) data, thus providing a compelling way to interpret causal effect measures (18). The random allocation of alleles can effectively regulate the traditional confounding factors to ensure that these factors are evenly distributed in different genotypes. In addition, MR studies exclude the possibility of reverse causality. The use of MR allows genetic variation to substitute for modifiable variables like exposure factors. A causal genetic association between exposure and outcome can be inferred using natural genetic variation. MR facilitates the discovery of causal relationships between diseases, and it has become a valuable tool in epidemiology and genetics.

Numerous studies have used MR to explore a causal association between mental disorders and other diseases. For example, some scholars have found that major depressive disorder was associated with a higher sleep apnea risk (19). Some scholars have found an increased risk of non-glioblastoma multiforme glioma in patients with schizophrenia (20). Other studies have found that depression may increase the susceptibility of women to infertility (21). Furthermore, some studies have found that atopic dermatitis can increase the risk of autism spectrum disorder, suggesting a potential causal association between the two (22). However, to our knowledge, no MR studies exploring a genetic causal link between mental illness and TB have been conducted. Using GWAS data, we conducted bidirectional two-sample MR (TSMR) analyses for some mental disorders (major depressive disorder, anxiety, bipolar disorder, and schizophrenia) and TB to provide new ideas for global public health prevention and control.

## 2 Materials and methods

### 2.1 Study design

**Figure 1:**
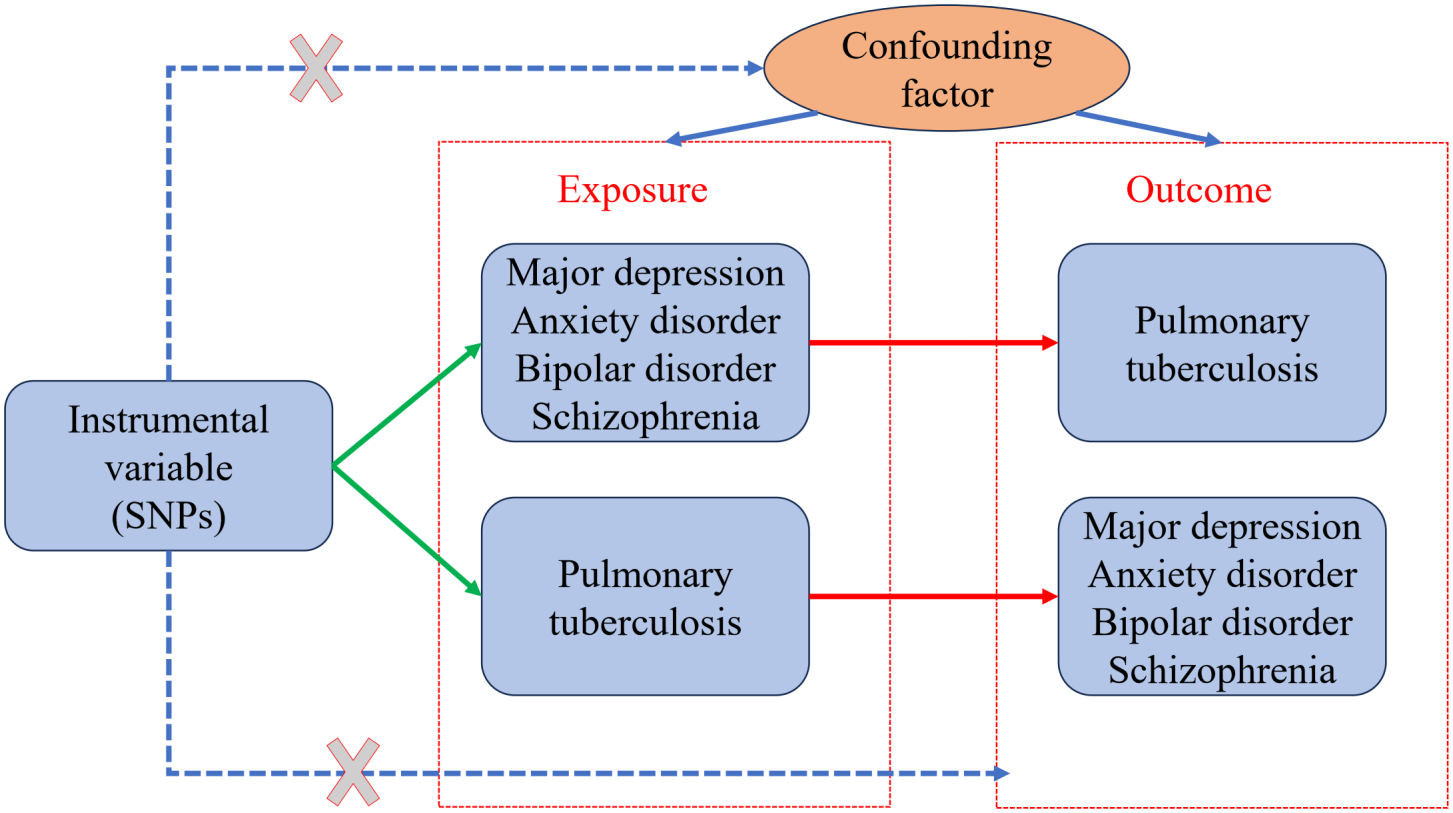
Two-sample bidirectional Mendelian randomization (MR) framework and assumptions of mental illness and pulmonary tuberculosis.

A bidirectional TSMR study was conducted to examine the causal genetic association between mental illness and pulmonary TB using the selected instrumental variables. The study design is described in a flowchart (**Figure 1**). A valid MR analysis needs to satisfy the following key assumptions: (1) To be an instrumental variable, a genetic variant must be highly associated with exposure; (2) The exposure–outcome relationship should not be influenced by any confounding factor for genetic variants; and (3) Exposure is the only way for genetic variants to impact outcomes, without horizontal pleiotropic effects. Valid SNPs must be screened as instrumental variables for subsequent studies through an MR study. The instrumental variables that were screened first must have a strong association with mental illness. In addition, the instrumental variables must be unaffected by any factors associated with mental illness and TB. Finally, the screened instrumental variables had to affect TB through mental illness. For reverse MR, it is necessary to have an instrumental variable that is highly associated with pulmonary TB. Any confounding factors related to mental illness or TB should not affect the instrumental variables. It was determined that the instrumental variables screened were associated with mental illness through TB. No additional ethical approval or informed consent was required because the data were retrieved from public databases.

### 2.2 Data source

GWAS datasets are a reliable tool for MR analysis. In our study, genetic associations of SNPs with major depressive were determined using the biggest published GWAS meta-analysis for European ancestry. It includes the UK Biobank and PGC datasets (170,756 cases, 329,443 controls), excluding 23andme (23). Summary data are available from the IEU OPEN GWAS website (GWAS ID: ieu-b-102). Bipolar disorder-related data were derived from a GWAS meta-analysis of the PGC Bipolar Disorder Working Group of the Psycho Genomics Consortium. Bipolar disorder-related data included 57 bipolar disorder cohorts of European ancestry (41,917 cases, 371,549 controls) from Europe, North America, and Australia (24). Summary data are available from the IEU OPEN GWAS website (GWAS ID: ieu-b-5110). Schizophrenia-related data were also obtained from the PCG database, which included 52,017 cases and 75,889 controls (25). Summary data are available from the IEU OPEN GWAS website (GWAS ID: ieu-b-5102). Data pertaining to anxiety disorders were obtained from the Neale lab analysis of UK Biobank phenotypes, including 16,730 cases and 101,021 controls. Its summary data can be obtained from the IEU OPEN GWAS website (GWAS ID: ukb-d-20544_15). The TB case summary data were obtained from the IEU OPEN GWAS website (GWAS ID: finn-b-TBC_RESP) and included 849 TB cases and 217,632 controls. All data on the discussed mental illnesses and pulmonary TB were obtained for individuals of European ancestry, and the sample populations did not overlap.

### 2.3 Selection of the genetic instruments

We developed a quality control step to identify instrumental variables (IVs) that met the MR analysis assumptions. First, SNPs of instrumental variables were found to be strongly associated with mental illness (major depressive, bipolar disorder, and schizophrenia). A *P* value of <5 × 10^-8^ was set as the significance threshold to obtain the SNPs. As the SNPs related to anxiety disorder were fewer than 3, we used the genetic instruments on a lower significance threshold (*P* < 5 × 10^-6^) to get more SNPs. Second, we performed clustering to eliminate SNPs with significant linkage disequilibrium (LD) by setting the R2 threshold at 0.001 and the window size at 10,000 kb. Third, SNPs with minor allele frequencies <0.01 were not included. Fourth, when SNPs with high LD (r2 > 0.8) were not found in the outcome GWAS, these SNPs were replaced by target SNPs. Finally, to ensure that the effect alleles were of the same allele, by comparing the exposure and outcome datasets, we were able to identify ambiguous SNPs with discrepancies in allele frequencies and ambiguous SNPs with intermediate allele frequencies. The final genetic IVs for subsequent MR analyses were determined by carefully selecting these SNPs. Furthermore, the formula used by us for calculating the individual and cumulative F-statistics for each SNP was *F* = *R^2^* (*N*−2)/(1−*R^2^*) (26). *R^2^* is the proportion of variance in the phenotype explained by the genetic variants. *N* is the sample size of the GWAS for the SNP–physical activity association. The MR analysis did not include genetic IVs that have an F-statistic lower than 10, which are considered weak instruments. Finally, a reverse MR analysis was performed with the same procedure. As there were few SNPs with *P* values of <5 × 10^-8^ in TB, we expanded the threshold to 5 × 10^-6^ to select eligible instrumental variables.

### 2.4 Statistical analysis

In this study, to determine the potential causal associations between mental illness and pulmonary TB, the inverse variance weighting (IVW) method was the primary method used. A comprehensive evaluation of the possible relationship was made possible by the use of three effective methods: weighted median, MR–Egger, and weighted mode. Assuming all the selected genetic variants to be valid IVs without pleiotropy, weighted analysis was performed by the IVW method without considering the intercept using the inverse variance of the outcome, which can ideally provide an unbiased causality assessment (27). The weighted median method can yield accurate and robust estimates when >50% of the valid instrumental variables are available (28). An adaption of Egger regression (which we call MR-Egger) can detect some violations of the standard instrumental variable assumptions and provide an effect estimate which is not subject to these violations. The approach provides a sensitivity analysis for the robustness of the findings from an MR investigation (29). The validity of the largest subset of instruments with similar causal effects is necessary for the weighted mode to be reliable (30).

### 2.5 Pleiotropy and sensitivity analysis

First, we performed several sensitivity analyses. The Cochrane Q statistic was used to determine heterogeneity between SNPs; heterogeneity was considered to be absent when the *P* value was >0.05. Second, the MR–Egger regression was used to evaluate gene pleiotropy-induced bias. A *P* value of >0.05 indicated the absence of horizontal pleiotropy. Third, to identify where outliers had a significant effect on the results, we used leave-one-out analysis, which involved eliminating each SNP sequentially and then performing IVW on the remaining SNPs. Finally, the MR analysis results were visualized using scatter plots, forest plots, and leave‐one‐out analysis. We processed the data in R software (version 4.3.2) using the R package TwoSampleMR (31).

## 3 Results

### 3.1 Causal effects of mental illness on pulmonary tuberculosis

**Figure 2:**
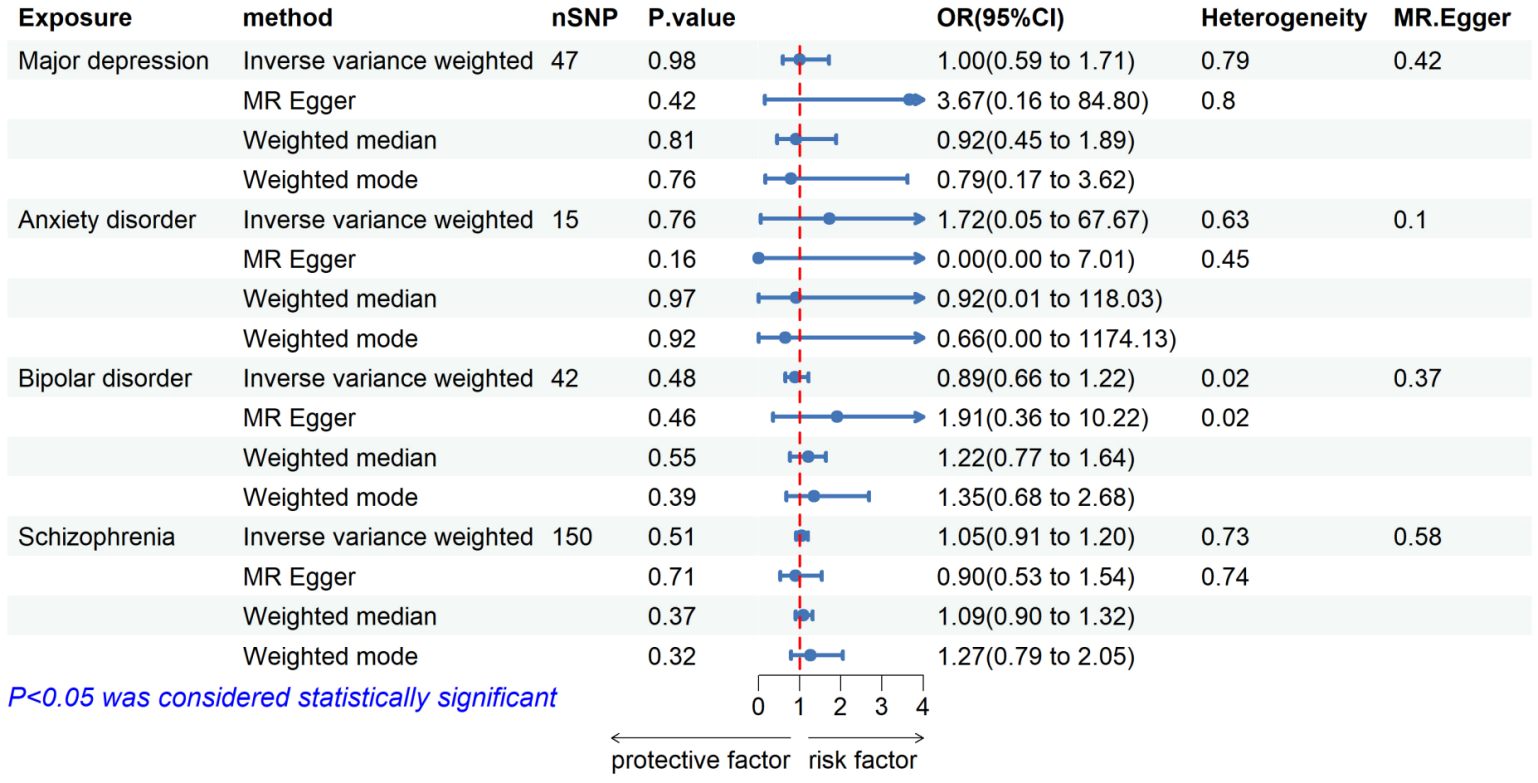
Forest plot showing MR results of the effect of mental illness on pulmonary tuberculosis.

We included 50, 17, 52, and 158 independent SNPs as genetic IVs for major depressive, anxiety disorder, bipolar disorder, and schizophrenia, respectively. **Supplementary Table 1** shows specific information about instrumental variables. Our MR study analysis showed no causal associations between any of the studied mental illnesses and pulmonary TB when mental illness characteristics were set as exposure, as detailed in **Figure 2**. The results of the IVW analysis showed that major depressive [odds ratio (OR) = 1.00, 95% confidence interval (CI) = 0.59–1.71, *P* = 0.98], anxiety disorder (OR = 1.72, 95% CI = 0.05–67.67, *P* = 0.76), bipolar disorder (OR = 0.89, 95% CI = 0.66–1.22, *P* = 0.48), and schizophrenia (OR = 1.05, 95% CI = 0.91–1.20, *P* = 0.51) were not causally related to the risk of pulmonary TB. The same results were obtained for weighted median, MR–Egger, and weighted mode. No heterogeneity among the SNPs was noted using the Cochrane Q test. The MR–Egger regression results showed no horizontal pleiotropy. Leave-one-out analyses showed that none of the SNPs had a substantial effect on the causal estimates of the relationship between mental illnesses and pulmonary TB. The findings of the leave-one-out analyses are shown in **Supplementary Figure 1**.

### 3.2 Causal effects of pulmonary tuberculosis on mental illness

**Figure 3:**
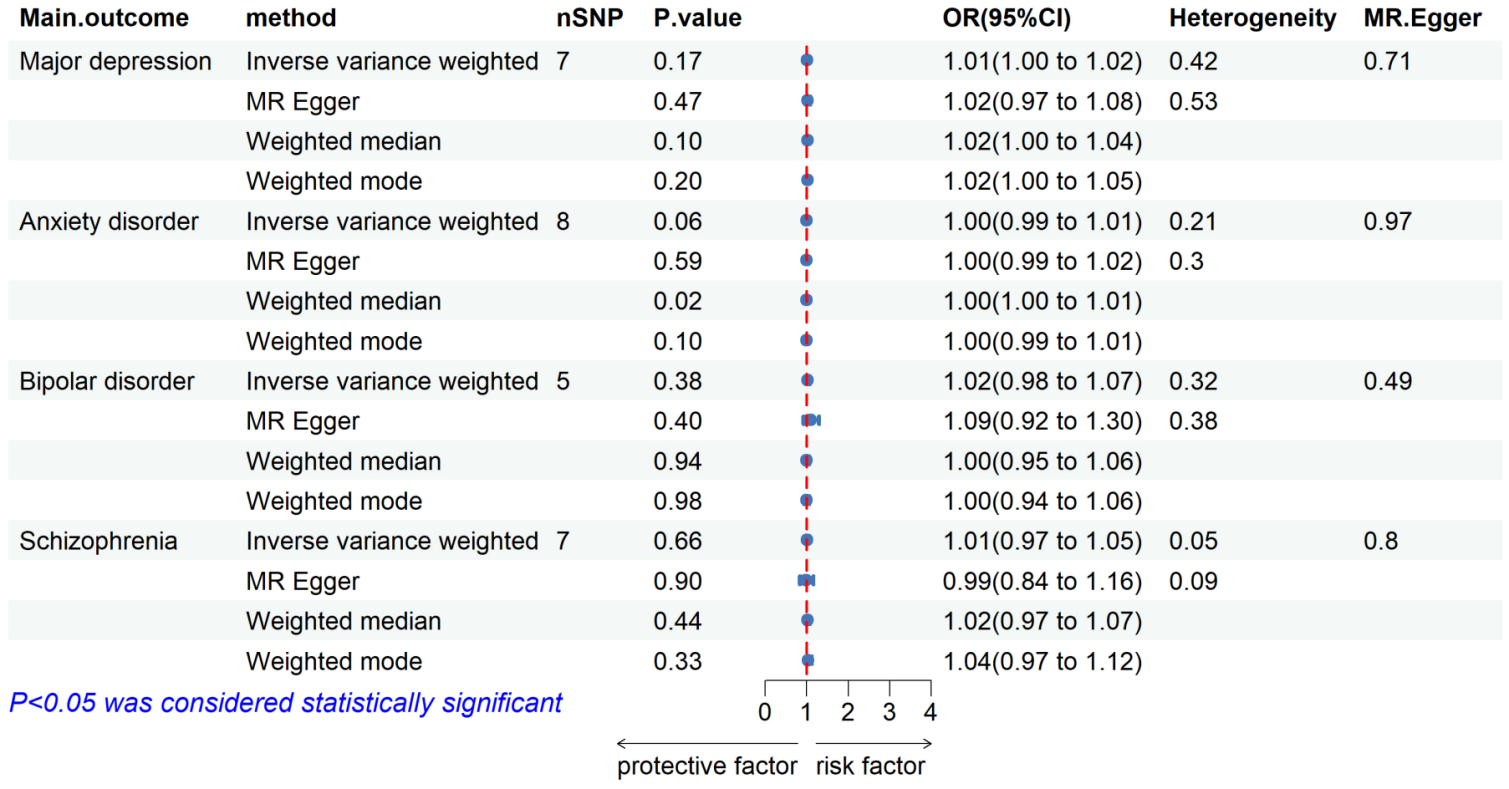
Forest plot showing MR results of the effect of pulmonary tuberculosis on mental illness.

We extracted 10 independent SNPs with P values of <5 × 10^-6^ as genetic IVs when using pulmonary TB characteristics as exposures. Details about these IVs are provided in **Supplementary Table 2**. TB was not found to be causally associated with any of the studied mental illnesses, as shown in **Figure 3**. Based on the IVW method, no significant association was found between pulmonary TB and the risk of mental illness (major depressive: OR = 1.01, 95% CI = 1.00–1.02, *P* = 0.17; anxiety disorder: OR = 1.00, 95% CI = 0.99–1.01, *P* = 0.06; bipolar disorder: OR = 1.02, 95% CI = 0.98–1.07, *P* = 0.38; and schizophrenia: OR = 1.01, 95% CI = 0.97–1.05, *P* = 0.66). (**Supplementary Figure 2**).

## 4 Discussion

Earlier epidemiological studies found an association between psychological illness and TB. A meta-analysis in East Africa showed that the prevalence of depression in TB patients was 43.03%; specifically, Kenya had the highest prevalence of depression in TB patients at 45.71%, followed by Ethiopia at 45.11% (32). A recent meta-analysis on TB multimorbidity in low- and middle-income countries also revealed depression as the most common comorbidity among pulmonary TB patients, affecting 45.19% of patients (33). In a cohort study of latent pulmonary TB, major depressive and anxiety had prevalence rates of 40% and 48%, respectively (34). Another meta-analysis found that depression and schizophrenia increase the risk of active TB (35). Numerous studies seem to indicate a causal link between the two diseases.

The reason underlying the association between mental illness and TB may be related to immunity. The immune system consists of biological structures and processes that help the body cope with physical or psychological stress. In the presence of physical injury or significant psychological stress, the inflammatory response of the affected area increases, and many innate immune cells migrate to the area as a defense mechanism (36). The activation of these innate immune mechanisms, including proinflammatory cytokines, such as IL-1, IL-6, TNF-α, and CRP, may contribute to the development of psychiatric disorders (14, 35, 37, 38). In addition to increased innate immune response, cell-mediated adaptive immune activation also occurs in patients with depression, and one of the key features of this activation is increased CD4^+^/CD8^+^ T cells ratio (39). Besides the enhanced immune response in response to pathogens, T cell responses can also be weakened in patients with mental illness (40). It has been suggested that depression is associated with changes in the immune system, both in terms of activation and suppression of the immune system. Another recently conducted meta-analysis has suggested that the development of depression involves innate and adaptive immunity as well as neuroendocrine circuits (41). Exposure to external bacterial or viral components, exercise, and psychological or physiological stress due to trauma are some of the factors that can cause inflammation. Consequently, these triggers activate immune responses and induce the production of proinflammatory cytokines, chemokines, and acute-phase proteins. The interactions between innate and adaptive immune cells, inflammatory factors, and neuroendocrine molecules in the peripheral and central nervous systems profoundly impact the development of depression. In addition, it has been discovered that inflammatory states may also lead to the abnormal activation of microglia and the release of cytokines, resulting in an imbalance of dopaminergic, serotonergic, and glutamate neurotransmission. Consequently, this can induce schizophrenia in susceptible individuals (37).

Immune dysfunction is also the primary cause of TB progression. Studies have shown that the inflammatory cytokine IL-1 plays a significant role in the regulation of MTB infection. Reduced IL-1 responses and increased type I IFN production were found to be associated with the worsening of TB disease in both mice and human patients infected with TB (15). In depression, the excessive production of IL-1β negatively impacts the body’s inflammatory response and can hasten the progression of TB. Moreover, the concentration of TNF-α and IL-1β in the lungs is directly correlated with the extent of lung involvement and the size of tuberculous cavities (42). Similarly, other studies have found excessive inflammation or immune dysfunction to be harmful to the host as it promotes the entry of additional MTB into cells and the subsequent cell death, thus exacerbating TB (43). In patients with major depressive disorder, disturbed phospholipid metabolism may contribute to inflammation in the central nervous system (44). Similarly, lipid degradation also plays an important role in maintaining MTB replication and intracellular survival (45). Some scholars have found that the Th1/Th2 imbalance in patients with major depressive can inhibit the host immune response to MTB (46).

Many studies have shown that mental illness and TB co-exist highly and increase each other’s incidence and have also provided mechanistic explanations for the association between mental illness and pulmonary TB. However, our analysis does not support a causal association between mental illness and TB. Notably, limited patient cohort size, recruitment bias, and heterogeneity of meta-analysis studies may have compromised the accuracy of the results of previous observational studies. For example. people with mental illnesses tend to have lower incomes and worse living conditions, which can make them more susceptible to TB. Similarly, patients with pulmonary TB are susceptible to discrimination from the surrounding population, unemployment, and poor prognosis of the disease, thus possibly leading to a mental illness. These confounding factors could not be corrected for in previous studies. There may be a correlation between the pathogenesis of the two diseases, but it is not sufficient to prove a causal genetic association.

The study is the first to examine bidirectional causal associations between mental illness and TB using multiple complementary MR techniques. Although a genetic association between mental illness and pulmonary TB seemed implied based on existing data of populations of European ancestry, we could not identify any causal associations between mental illness and pulmonary TB from a genetics perspective. We acknowledge the causal mechanisms that have been studied so far. To provide more conclusive results, it is necessary to conduct further analyses using larger datasets and implement additional quality control steps. The future availability of more data with the power to explain a larger portion of the phenotypic variance could yield more evidence of a causal relationship between mental illness and TB.

However, it is important to note that this MR study has some limitations. First, the population in this study was of European ancestry, and it remains unknown whether the findings would apply to other populations globally. Second, although the powerful tools that we have used in this study (F < 10 was ruled out) reduce any potential bias due to sample overlap, we cannot completely rule out the possibility of overlap of individuals in exposure and outcome studies. Third, although the results of sensitivity analyses are robust, horizontal pleiotropy of genetic variation cannot be completely ruled out. Fourth, the *P* value of the weighted median (WM) method in the MR analysis of TB and anxiety disorders was < 0.05, whereas the values for the other methods were >0.05. WM was the median of the distribution function of all individual SNP effect values ranked by weight. WM could obtain a robust estimate when at least 50% of the information came from valid instrumental variables. However, the premise of this study is that all SNPS are effective instrumental variables and completely independent of each other, and thus, the IVW method is preferred in this study. Although WM results can be used as a reference, they cannot replace the results of the IVW method. Finally, this is the first study on the causal relationship between mental illness and TB, and the sample size of the current GWAS data is limited.

## 5 Conclusion

In conclusion, our findings do not support a bidirectional causal association between the studied mental illnesses and pulmonary TB. More recent genetics-based MR analytical instruments and large-scale GWAS summary data are needed to validate the results of this study.

## Data availability statement

The original contributions presented in the study are included in the article/**Supplementary Material**. Further inquiries can be directed to the corresponding author.

## Ethics statement

Ethical approval was not required for the study involving humans in accordance with the local legislation and institutional requirements. Written informed consent to participate in this study was not required from the participants or the participants’ legal guardians/next of kin in accordance with the national legislation and the institutional requirements.

## Author contributions

XC: Writing – original draft, Writing – review & editing, Data curation, Methodology. FY: Formal analysis, Software, Supervision, Writing – review & editing. RH: Software, Validation, Visualization, Writing – review & editing.
