# Supplementary figures and images for: Mental illness and pulmonary tuberculosis: a bidirectional two-sample Mendelian randomization study
Source: Front Psychiatry. 2024 Apr 29;15:1345863. doi: 10.3389/fpsyt.2024.1345863 (PMC11089237; doi:10.3389/fpsyt.2024.1345863)

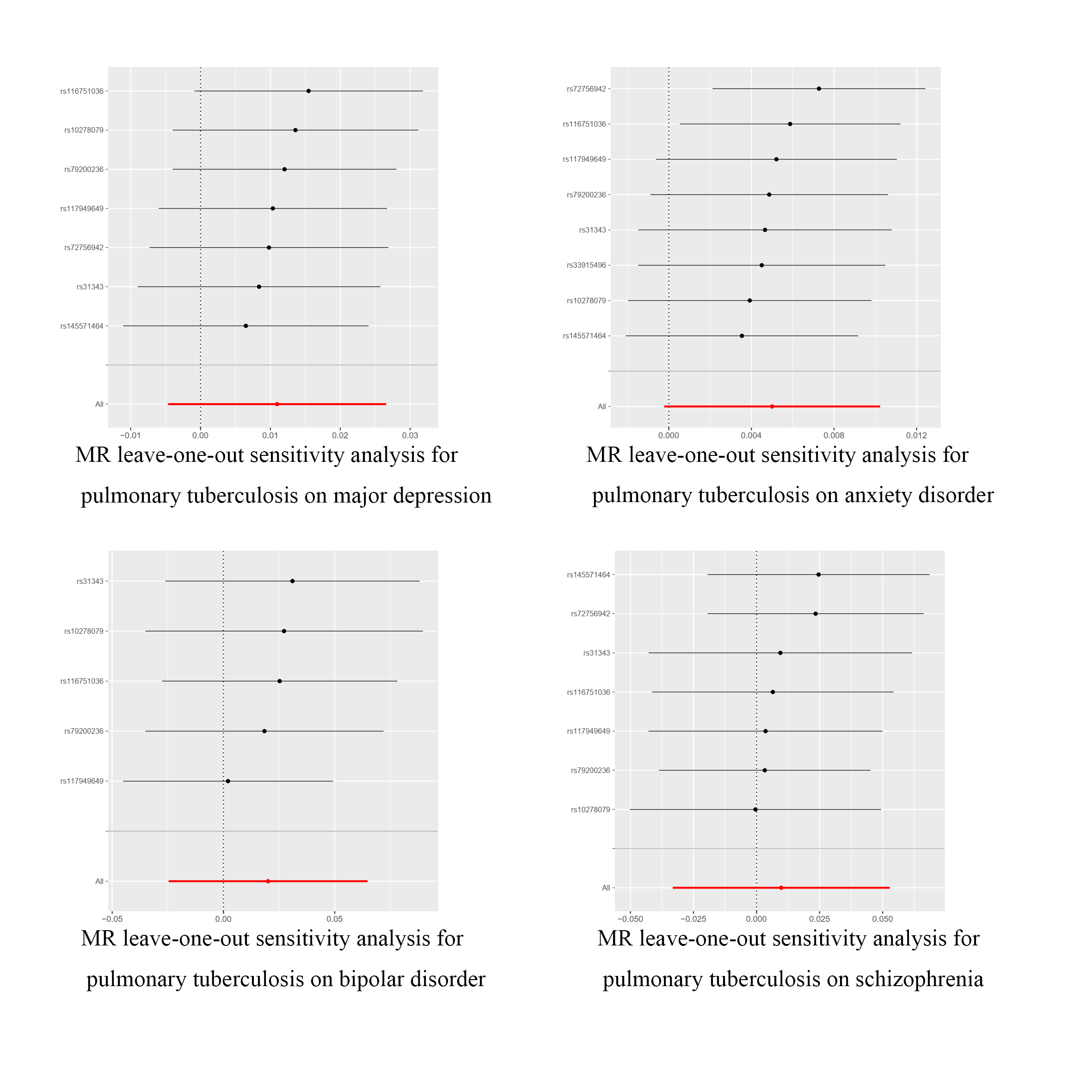

Supplement: Supplementary file 3 [file Image_1.tif]

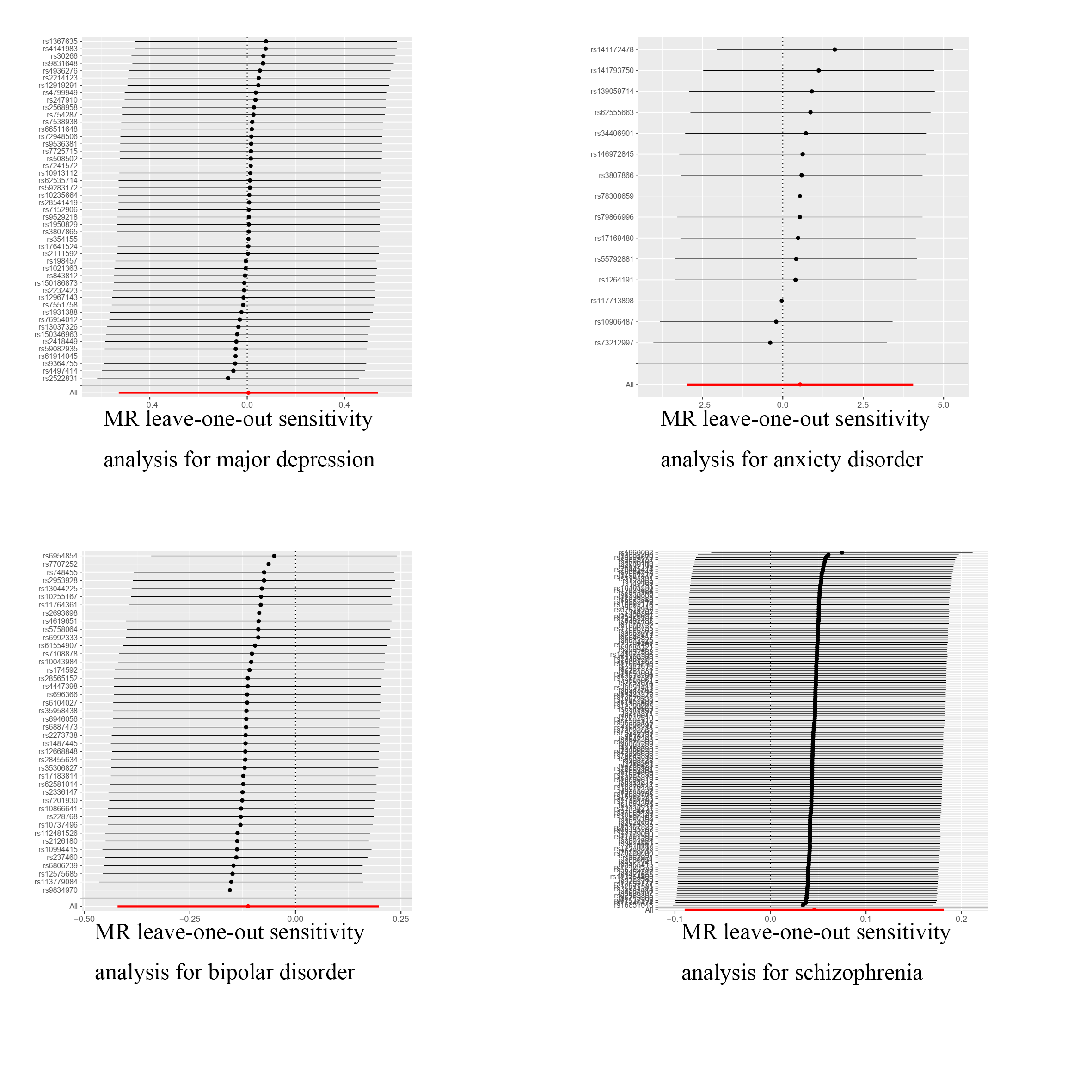

Supplement: Supplementary file 4 [file Image_2.tif]
